# Supplementary material for: Differences in stiffness across the patellar tendon: An observational study using tendotonometry
Source: PLoS One. 2025 Sep 17;20(9):e0329710. doi: 10.1371/journal.pone.0329710 (PMC12443289; doi:10.1371/journal.pone.0329710)
Supplement: S1 Table — (DOCX) [file pone.0329710.s001.docx]

**Table S1. Sports performed by participants**

| **Sport** | **Males** | **Females** |
| --- | --- | --- |
| Basketball | 1 | 0 |
| Climbing | 2 | 1 |
| Cycling | 3 | 2 |
| Fitness | 7 | 8 |
| Frisbee | 1 | 0 |
| Hockey | 1 | 1 |
| Kickboxing | 1 | 0 |
| Lacrosse | 1 | 4 |
| Pole dancing | 0 | 1 |
| Rowing | 1 | 1 |
| Running | 2 | 4 |
| Skating | 2 | 1 |
| Soccer | 2 | 0 |
| Volleyball | 0 | 1 |
