# Supplementary material for: Differences in stiffness across the patellar tendon: An observational study using tendotonometry
Source: PLoS One. 2025 Sep 17;20(9):e0329710. doi: 10.1371/journal.pone.0329710 (PMC12443289; doi:10.1371/journal.pone.0329710)
Supplement: S2 Table — 1) proximal-medial, 2) proximal-horizontal midline, 3) proximal-lateral, 4)vertical midline-medial, 5) vertical midline-horizontal midline, 6) vertical midline-lateral, 7) distal-medial, 8) distal- horizontal midline, 9) distal-lateral. For the right knee, medial and lateral are reversed. (DOCX) [file pone.0329710.s002.docx]

**Table S2a. Tukey HSD posthoc comparisons of the nine different measurement locations within the female patellar tendon**

|  |  |  |  | **95% CI** | | |
| --- | --- | --- | --- | --- | --- | --- |
| **Location** | **Compared to location** | **Mean difference** | **p-value** | **Lower bound** | **Upper bound** | |
| 1 | 2 | -143.38* | .003 | -256.36 | -30.40 | |
|  | 3 | -76.01 | .470 | -188.99 | | 36.96 |
|  | 4 | 36.92 | .983 | -76.06 | | 149.89 |
|  | 5 | -65.05 | .679 | -178.02 | | 47.93 |
|  | 6 | 28.08 | .997 | -84.90 | | 141.06 |
|  | 7 | -43.87 | .952 | -156.85 | | 69.11 |
|  | 8 | -86.63 | .288 | -199.60 | | 26.35 |
|  | 9 | -31.97 | .993 | -144.95 | | 81.01 |
| 2 | 1 | 143.38* | .003 | 30.40 | | 256.36 |
|  | 3 | 67.37 | .636 | -45.61 | | 180.34 |
|  | 4 | 180.30* | .000 | 67.32 | | 293.27 |
|  | 5 | 78.33 | .427 | -34.64 | | 191.31 |
|  | 6 | 171.46* | .000 | 58.48 | | 284.43 |
|  | 7 | 99.51 | .134 | -13.47 | | 212.48 |
|  | 8 | 56.75 | .817 | -56.22 | | 169.73 |
|  | 9 | 111.41 | .057 | -1.57 | | 224.38 |
| 3 | 1 | 76.01 | .470 | -36.96 | | 188.99 |
|  | 2 | -67.37 | .636 | -180.34 | | 45.61 |
|  | 4 | 112.93 | .050 | -.05 | | 225.91 |
|  | 5 | 10.97 | 1.000 | -102.01 | | 123.94 |
|  | 6 | 104.09 | .098 | -8.88 | | 217.07 |
|  | 7 | 32.14 | .993 | -80.83 | | 145.12 |
|  | 8 | -10.61 | 1.000 | -123.59 | | 102.36 |
|  | 9 | 44.04 | .951 | -68.93 | | 157.02 |
| 4 | 1 | -36.92 | .983 | -149.89 | | 76.06 |
|  | 2 | -180.30* | .000 | -293.27 | | -67.32 |
|  | 3 | -112.93 | .050 | -225.91 | | .05 |
|  | 5 | -101.96 | .113 | -214.94 | | 11.01 |
|  | 6 | -8.84 | 1.000 | -121.81 | | 104.14 |
|  | 7 | -80.79 | .383 | -193.76 | | 32.19 |
|  | 8 | -123.54* | .021 | -236.52 | | -10.57 |
|  | 9 | -68.89 | .607 | -181.86 | | 44.09 |
| 5 | 1 | 65.05 | .679 | -47.93 | | 178.02 |
|  | 2 | -78.33 | .427 | -191.31 | | 34.64 |
|  | 3 | -10.97 | 1.000 | -123.94 | | 102.01 |
|  | 4 | 101.96 | .113 | -11.01 | | 214.94 |
|  | 6 | 93.13 | .200 | -19.85 | | 206.10 |
|  | 7 | 21.18 | 1.000 | -91.80 | | 134.15 |
|  | 8 | -21.58 | 1.000 | -134.56 | | 91.40 |
|  | 9 | 33.08 | .992 | -79.90 | | 146.05 |
| 6 | 1 | -28.08 | .997 | -141.06 | | 84.90 |
|  | 2 | -171.46* | .000 | -284.43 | | -58.48 |
|  | 3 | -104.09 | .098 | -217.07 | | 8.88 |
|  | 4 | 8.84 | 1.000 | -104.14 | | 121.81 |
|  | 5 | -93.13 | .200 | -206.10 | | 19.85 |
|  | 7 | -71.95 | .548 | -184.93 | | 41.03 |
|  | 8 | -114.70* | .044 | -227.68 | | -1.73 |
|  | 9 | -60.05 | .766 | -173.03 | | 52.93 |
| 7 | 1 | 43.87 | .952 | -69.11 | | 156.85 |
|  | 2 | -99.51 | .134 | -212.48 | | 13.47 |
|  | 3 | -32.14 | .993 | -145.12 | | 80.83 |
|  | 4 | 80.79 | .383 | -32.19 | | 193.76 |
|  | 5 | -21.18 | 1.000 | -134.15 | | 91.80 |
|  | 6 | 71.95 | .548 | -41.03 | | 184.93 |
|  | 8 | -42.75 | .959 | -155.73 | | 70.22 |
|  | 9 | 11.90 | 1.000 | -101.08 | | 124.88 |
| 8 | 1 | 86.63 | .288 | -26.35 | | 199.60 |
|  | 2 | -56.75 | .817 | -169.73 | | 56.22 |
|  | 3 | 10.61 | 1.000 | -102.36 | | 123.59 |
|  | 4 | 123.54* | .021 | 10.57 | | 236.52 |
|  | 5 | 21.58 | 1.000 | -91.40 | | 134.56 |
|  | 6 | 114.70* | .044 | 1.73 | | 227.68 |
|  | 7 | 42.75 | .959 | -70.22 | | 155.73 |
|  | 9 | 54.65 | .847 | -58.32 | | 167.63 |
| 9 | 1 | 31.97 | .993 | -81.01 | | 144.95 |
|  | 2 | -111.41 | .057 | -224.38 | | 1.57 |
|  | 3 | -44.04 | .951 | -157.02 | | 68.93 |
|  | 4 | 68.89 | .607 | -44.09 | | 181.86 |
|  | 5 | -33.08 | .992 | -146.05 | | 79.90 |
|  | 6 | 60.05 | .766 | -52.93 | | 173.03 |
|  | 7 | -11.90 | 1.000 | -124.88 | | 101.08 |
|  | 8 | -54.65 | .847 | -167.63 | | 58.32 |
|  |  |  |  |  |  |  |

1) proximal-medial, 2) proximal-horizontal midline, 3) proximal-lateral, 4)vertical midline-medial, 5) vertical midline-horizontal midline, 6) vertical midline-lateral, 7) distal-medial, 8) distal- horizontal midline, 9) distal-lateral. For the right knee, medial and lateral are reversed.
