# Supplementary material for: Differences in stiffness across the patellar tendon: An observational study using tendotonometry
Source: PLoS One. 2025 Sep 17;20(9):e0329710. doi: 10.1371/journal.pone.0329710 (PMC12443289; doi:10.1371/journal.pone.0329710)
Supplement: S3 Table — 1) proximal-medial, 2) proximal-horizontal midline, 3) proximal-lateral, 4)vertical midline-medial, 5) vertical midline-horizontal midline, 6) vertical midline-lateral, 7) distal-medial, 8) distal- horizontal midline, 9) distal-lateral. For the right knee, medial and lateral are reversed. (DOCX) [file pone.0329710.s003.docx]

**Table S2b. Tukey HSD posthoc comparisons of the nine different measurement locations within the male patellar tendon**

|  |  |  |  | **95% CI** | | |
| --- | --- | --- | --- | --- | --- | --- |
| **Location** | **Compared to location** | **Mean difference** | **p-value** | **Lower bound** | **Upper bound** | |
| 1 | 2 | -125,58* | ,004 | -226,85 | -24,31 | |
|  | 3 | -33,34 | ,982 | -134,61 | | 67,93 |
|  | 4 | 32,68 | ,984 | -68,60 | | 133,95 |
|  | 5 | -58,59 | ,673 | -159,86 | | 42,69 |
|  | 6 | 42,08 | ,930 | -59,20 | | 143,35 |
|  | 7 | -31,37 | ,988 | -132,65 | | 69,90 |
|  | 8 | -59,50 | ,654 | -160,77 | | 41,77 |
|  | 9 | 10,69 | 1,000 | -90,59 | | 111,96 |
| 2 | 1 | 125,58* | ,004 | 24,31 | | 226,85 |
|  | 3 | 92,24 | ,106 | -9,04 | | 193,51 |
|  | 4 | 158,25* | <,001 | 56,98 | | 259,53 |
|  | 5 | 66,99 | ,494 | -34,28 | | 168,26 |
|  | 6 | 167,65* | <,001 | 66,38 | | 268,93 |
|  | 7 | 94,20 | ,091 | -7,07 | | 195,48 |
|  | 8 | 66,08 | ,514 | -35,19 | | 167,35 |
|  | 9 | 136,27* | ,001 | 34,99 | | 237,54 |
| 3 | 1 | 33,34 | ,982 | -67,93 | | 134,61 |
|  | 2 | -92,24 | ,106 | -193,51 | | 9,04 |
|  | 4 | 66,02 | ,515 | -35,26 | | 167,29 |
|  | 5 | -25,25 | ,997 | -126,52 | | 76,03 |
|  | 6 | 75,42 | ,327 | -25,86 | | 176,69 |
|  | 7 | 1,97 | 1,000 | -99,31 | | 103,24 |
|  | 8 | -26,16 | ,996 | -127,43 | | 75,11 |
|  | 9 | 44,03 | ,910 | -57,24 | | 145,30 |
| 4 | 1 | -32,68 | ,984 | -133,95 | | 68,60 |
|  | 2 | -158,25* | <,001 | -259,53 | | -56,98 |
|  | 3 | -66,02 | ,515 | -167,29 | | 35,26 |
|  | 5 | -91,26 | ,114 | -192,54 | | 10,01 |
|  | 6 | 9,40 | 1,000 | -91,87 | | 110,67 |
|  | 7 | -64,05 | ,557 | -165,32 | | 37,22 |
|  | 8 | -92,18 | ,107 | -193,45 | | 9,10 |
|  | 9 | -21,99 | ,999 | -123,26 | | 79,29 |
| 5 | 1 | 58,59 | ,673 | -42,69 | | 159,86 |
|  | 2 | -66,99 | ,494 | -168,26 | | 34,28 |
|  | 3 | 25,25 | ,997 | -76,03 | | 126,52 |
|  | 4 | 91,26 | ,114 | -10,01 | | 192,54 |
|  | 6 | 100,66 | ,053 | -,61 | | 201,94 |
|  | 7 | 27,21 | ,995 | -74,06 | | 128,49 |
|  | 8 | -,91 | 1,000 | -102,19 | | 100,36 |
|  | 9 | 69,27 | ,446 | -32,00 | | 170,55 |
| 6 | 1 | -42,08 | ,930 | -143,35 | | 59,20 |
|  | 2 | -167,65* | <,001 | -268,93 | | -66,38 |
|  | 3 | -75,42 | ,327 | -176,69 | | 25,86 |
|  | 4 | -9,40 | 1,000 | -110,67 | | 91,87 |
|  | 5 | -100,66 | ,053 | -201,94 | | ,61 |
|  | 7 | -73,45 | ,363 | -174,72 | | 27,82 |
|  | 8 | -101,58* | ,049 | -202,85 | | -,30 |
|  | 9 | -31,39 | ,988 | -132,66 | | 69,89 |
| 7 | 1 | 31,37 | ,988 | -69,90 | | 132,65 |
|  | 2 | -94,20 | ,091 | -195,48 | | 7,07 |
|  | 3 | -1,97 | 1,000 | -103,24 | | 99,31 |
|  | 4 | 64,05 | ,557 | -37,22 | | 165,32 |
|  | 5 | -27,21 | ,995 | -128,49 | | 74,06 |
|  | 6 | 73,45 | ,363 | -27,82 | | 174,72 |
|  | 8 | -28,13 | ,994 | -129,40 | | 73,15 |
|  | 9 | 42,06 | ,930 | -59,21 | | 143,34 |
| 8 | 1 | 59,50 | ,654 | -41,77 | | 160,77 |
|  | 2 | -66,08 | ,514 | -167,35 | | 35,19 |
|  | 3 | 26,16 | ,996 | -75,11 | | 127,43 |
|  | 4 | 92,18 | ,107 | -9,10 | | 193,45 |
|  | 5 | ,91 | 1,000 | -100,36 | | 102,19 |
|  | 6 | 101,58* | ,049 | ,30 | | 202,85 |
|  | 7 | 28,13 | ,994 | -73,15 | | 129,40 |
|  | 9 | 70,19 | ,427 | -31,09 | | 171,46 |
| 9 | 1 | -10,69 | 1,000 | -111,96 | | 90,59 |
|  | 2 | -136,27* | ,001 | -237,54 | | -34,99 |
|  | 3 | -44,03 | ,910 | -145,30 | | 57,24 |
|  | 4 | 21,99 | ,999 | -79,29 | | 123,26 |
|  | 5 | -69,27 | ,446 | -170,55 | | 32,00 |
|  | 6 | 31,39 | ,988 | -69,89 | | 132,66 |
|  | 7 | -42,06 | ,930 | -143,34 | | 59,21 |
|  | 8 | -70,19 | ,427 | -171,46 | | 31,09 |
|  |  |  |  |  |  |  |

1) proximal-medial, 2) proximal-horizontal midline, 3) proximal-lateral, 4)vertical midline-medial, 5) vertical midline-horizontal midline, 6) vertical midline-lateral, 7) distal-medial, 8) distal- horizontal midline, 9) distal-lateral. For the right knee, medial and lateral are reversed.
