# Supplementary material for: Differences in stiffness across the patellar tendon: An observational study using tendotonometry
Source: PLoS One. 2025 Sep 17;20(9):e0329710. doi: 10.1371/journal.pone.0329710 (PMC12443289; doi:10.1371/journal.pone.0329710)
Supplement: S4 Table — (DOCX) [file pone.0329710.s004.docx]

**Table S3a. ICC values (95%CI) of the threefold measured stiffness specified for females.**

| Location | Medial | Vertical midline | Lateral |
| --- | --- | --- | --- |
| Proximal | 0.970 (0.941-0.986) | 0.967 (0.937-0.985) | 0.973 (0.947-0.987) |
| Horizontal midline | 0.975 (0.952-0.988) | 0.989 (0.978-0.995) | 0.967 (0.937-0.985) |
| Distal | 0.966 (0.932-0.984) | 0.985 (0.971-0.993) | 0.956 (0.916-0.979) |
