# Supplementary material for: Differences in stiffness across the patellar tendon: An observational study using tendotonometry
Source: PLoS One. 2025 Sep 17;20(9):e0329710. doi: 10.1371/journal.pone.0329710 (PMC12443289; doi:10.1371/journal.pone.0329710)
Supplement: S5 Table — (DOCX) [file pone.0329710.s005.docx]

**Table S3b.** **ICC values (95%CI) of the threefold measured stiffness specified for males.**

| Location | Medial | Vertical midline | Lateral |
| --- | --- | --- | --- |
| Proximal | 0.927 (0.861-0.966) | 0.977 (0.956-0.989) | 0.950 (0.904-0.976) |
| Horizontal midline | 0.953 (0.904-0.979) | 0.986 (0.971-0.994) | 0.982 (0.964-0.992) |
| Distal | 0.974 (0.950-0.988) | 0.980 (0.960-0.991) | 0.955 (0.915-0.979) |
