# Supplementary material for: Differences in stiffness across the patellar tendon: An observational study using tendotonometry
Source: PLoS One. 2025 Sep 17;20(9):e0329710. doi: 10.1371/journal.pone.0329710 (PMC12443289; doi:10.1371/journal.pone.0329710)
Supplement: Attachment — Submitted filename: PONE-D-25-19089 comments.docx [file pone.0329710.s007.docx]

Thank you for the opportunity to review this manuscript.

1. Overall, this manuscript could use a significant amount of work to increase readability to bring it to the scientific standard. Please copy edit for grammar, acronym usage, punctuation, formatting (tables, spacing, references), and writing style (overly verbose at times).
2. I can see the usefulness of devices like the MyotonPRO as a method of quickly assessing a mechanical value of the tendon in a reliable manner. However, when we think of stiffness of a tendon, we typically think of it with regards to the tensile properties along the long axis in line with the collagen fibres/fascicles. I acknowledge that the authors do note that this is a form of compressive stiffness and make mention of the questions surrounding validity of the device in the limitations. Tendons are anisotropic tissues; and currently, we aren’t certain how compressive/transverse measures of stiffness relates to how tendons withstand tensile deformation. The authors should comment on this and add more of a distinction between the two mechanical measures in the paper - perhaps in the methods section and broaden the limitations statement as well.
3. The use of the term "tendotonomtry" is a neologism that is not found in the broader literature beyond the author’s previous publications. I recommend using more widely accepted terminology unless a strong justification is provided. Introducing new terms should be reserved for clearly novel concepts, and their definitions and distinctions from existing methods should be clearly articulated.
4. The authors need to expand on the gap in knowledge in the introduction, why is it important to take measurements in different locations on the PT? Why is it important to distinguish between recreational athletes and non-athletes? Also Zhu 2022 did not include females in their analysis, you could highlight that this is also part of the gap in knowledge.
5. The hypothesis needs to be clearly stated.
6. Line 80-83. “Measurements took place before any sports activities were performed by the athletes.” For that specific day? If the participants exercised, how long after exercise would the researchers wait to take measurements, were participants instructed not to exercise for x-hours/days prior to testing?
7. Line 93. What medications were a part of your exclusion criteria? Does this include medications like oral contraceptives, which have been shown to alter tendon mechanics?
8. How did you determine your sample size? Please provide necessary information.
9. Line 92. Given units for BMI (kg/m^2^). Was the stated BMI range an exclusion criteria? If yes, 25 kg/m^2^ is a very low cut-off value for BMI even in a healthy population. Please explain.
10. Lines 139-142 should be before “statistical significance was set at p<0.05”, lines 137-138.
11. Lines 145-146, “Males and females were combined together, since literature showed no difference in gender with respect to reliability of the measurement [50].” The authors could provide ICC analysis for both sexes, since the previous publication did not assess different locations on the PT and you’ve already provided some separate analysis of sex. When assessing biological differences, use sex rather than gender.
12. Table 2. Provide units, identify acronyms, tests used. Please report on the statistical test results. Publications have obligations to provide as much detail as possible so that your results can (potentially) be used for future reviews and field dissemination.
13. Lines 160-163. Same as comment #1, need some edits for readability especially when acronyms are being used. Example: both proximal and distal stiffness were greater than HM, and VM stiffness was greater than medial and lateral.
14. Table S2 could and probably should be presented as Table 3 within the results rather than supporting information. Also recheck ICC values, line 168 states all values were above 0.963, in the table it is 0.962. Perhaps a typo?
15. Lines 169 to 170. This is a little misleading, all cases were not <3%; in S3 you state a % of cases >3%. More clarity needed here. I also suggest adding S3 to the main body of the paper as well, or at least combining it with the previous table?
16. Lines 172-176. Once hypothesis is clarified (#5), a statement here would be appropriate.
17. Lines 188-189, broaden argument about why recreational athletes would have altered stiffness compared to non-athletes.
18. Line 150 “Maximum CV allowed for tendotonometry was preset at 3%”. I assume the authors would retake the exam if the measure was >3%? This adds confusion to lines 224-225 “mean CV below 3%”, the mean CV is always going to be below 3% because of the cut off value. There needs to be more clarity regarding CV and the 3% threshold.
19. General comment, there are a few statements throughout the manuscript that are overly definitive with regards to previous literature and your results; please make according adjustments.
