# Supplementary material for: Differences in stiffness across the patellar tendon: An observational study using tendotonometry
Source: PLoS One. 2025 Sep 17;20(9):e0329710. doi: 10.1371/journal.pone.0329710 (PMC12443289; doi:10.1371/journal.pone.0329710)
Supplement: Attachment — Submitted filename: Response_to_reviewers_auresp_2.docx [file pone.0329710.s010.docx]

**Response to editor and referees of manuscript PONE-D-25-19089**

Differences in stiffness across the patellar tendon. An observational study using tendotonometry.

Dear editor,

Thank you for offering the opportunity to revise the manuscript entitled “Differences in stiffness across the patellar tendon. An observational study using tendotonometry.” (PONE-D-25-19089). We are thankful to the reviewers for their useful and constructive feedback, which, we believe, has further improved our manuscript.

Below you will find our reply to the comments, which have been incorporated into the revised manuscript. We are submitting the revised manuscript using tracked changes.

Thank you for your time and effort to re-evaluate our revised manuscript.

We look forward to your decision.

Yours sincerely,

Also on behalf of the co-authors,

Lotte van Dam, MSc

Reviewer #1:

**1. Since reviewing this paper, there is a new publication that doesn't find a relationship between tensile stiffness and the MyotonPRO's compressive stiffness (Relationship between compressive stiffness and tensile stiffness in the human Achilles tendon in vivo. Ishigaki, Tomonobu et al. Journal of Bodywork and Movement Therapies, Volume 42, 1073 - 1078). My last revision suggests referencing this paper as it allows the author to be a little more decisive with their language in lines 303-307.**

1. We thank the reviewer for this feedback to improve the manuscript with more scientific references. We have incorporated the mentioned study in our strength and limitations section, in lines 303-312: “*Finally, PT stiffness measurements using the MyotonPRO quantify tendon stiffness perpendicular to the PT, while the actual tendon and its stiffness run longitudinal. We are not certain how compressive, transverse measures of stiffness relate to the ability of tendons to withstand tensile deformation. Resent evidence might suggest that compressive stiffness measured using the MyotonPRO does not reflect the tendon’s tensile stiffness under load, as Ishigaki et al. (2025) found no significant relationship between the two measures in the achilles tendon [73]. However, as this study compared measurements in rest (compressive stiffness) with measurements during exercise (tensile stiffness) and these measurements took place in a different tendon, extrapolation to the PT should be done with caution. Nevertheless, this study is proof that care is necessary when drawing conclusions about longitudinal versus perpendicular tendon stiffness.*”
